# The Longer-Term Effects of a Single Bupivacaine Exposure on the Mechanical Properties of Native Cartilage Explants

**DOI:** 10.1177/19476035231164751

**Published:** 2023-03-29

**Authors:** Kylie T. Callan, Gaston Otarola, Wendy E. Brown, Kyriacos A. Athanasiou, Dean Wang

**Affiliations:** 1 University of California, Irvine School of Medicine, Irvine, CA, USA; 2 Department of Biomedical Engineering, University of California, Irvine, Irvine, CA, USA; 3 Department of Orthopaedic Surgery, University of California Irvine Health, Orange, CA, USA

**Keywords:** cartilage, bupivacaine, mechanics, viability

## Abstract

**Objective:**

The purpose of this study was to determine the *in vitro* effects of a single exposure of bupivacaine on the mechanical properties of bovine cartilage explants at 3 weeks.

**Design:**

Femoral condyle articular cartilage explants were aseptically harvested from juvenile bovine stifle joints before being exposed to chondrogenic medium containing 0.50% (wt/vol) bupivacaine, 0.25% (wt/vol) bupivacaine, or no medication (control) for 1 hour. Explants were then washed and maintained in culture *in vitro* for 3 weeks before testing. Cell viability, tensile and compressive mechanical properties, histological properties, and biochemical properties were then assessed.

**Results:**

Explants exhibited a dose-dependent decrease in mean tensile Young’s modulus with increasing bupivacaine concentration (9.86 MPa in the controls, 6.48 MPa in the 0.25% bupivacaine group [*P* = 0.048], and 4.72 MPa in the 0.50% bupivacaine group [*P* = 0.005]). Consistent with these results, collagen content and collagen crosslinking decreased with bupivacaine exposure as measured by mass spectrometry. Compressive properties of the explants were unaffected by bupivacaine exposure. Explants also exhibited a trend toward dose-dependent decreases in viability (51.2% for the controls, 47.3% for the 0.25% bupivacaine-exposed group, and 37.0% for the 0.50% bupivacaine-exposed group [*P* = 0.072]).

**Conclusions:**

Three weeks after 1-hour bupivacaine exposure, the tensile properties of bovine cartilage explants were significantly decreased, while the compressive properties remained unaffected. These decreases in tensile properties corresponded with reductions in collagen content and crosslinking of collagen fibers. Physicians should be judicious regarding the intra-articular administration of bupivacaine in native joints.

## Introduction

Bupivacaine and other local anesthetics are often administered intra-articularly in healthy joints despite evidence demonstrating their short-term chondrotoxic effects. *In vitro* studies have shown that a 1-hour 0.50% bupivacaine exposure reduced the viability of cartilage explants to 16% to 65% 24 hours after exposure.^1,2^ In a dog model, 30 minutes of exposure to a 0.50% bupivacaine solution led to 53.8% chondrocyte death versus 20% chondrocyte death in control samples exposed only to chondrogenic medium.^3^ In a systematic review examining the effects of bupivacaine on human cartilage or chondrocytes, 13 out of 14 studies found it to be chondrotoxic.^4^ Evidence suggests that bupivacaine may be more chondrotoxic than other local anesthetics such as ropivacaine and mepivacaine.^1,5^

However, some studies have demonstrated no detrimental effect of bupivacaine exposure on long-term chondrocyte viability. For example, a rabbit model study found no effect of bupivacaine or bupivacaine in conjunction with epinephrine on viability 3 months after exposure, suggesting perhaps these chondrotoxic effects observed in the short-term may be transient.^6^ In a 2-part *in vitro* and *in vivo* donkey model, articular cartilage was exposed to 5% bupivacaine for 30 minutes, and outcomes demonstrated a viability of 97.3% and no differences in radiography, histopathology, or expression of cartilage catabolic marker genes.^7^

In another investigation, single bupivacaine exposure reduced the viability and weakened the mechanical properties of engineered neocartilage grafts at 24 hours.^2^ However, while the viability of mature cartilage explants was also reduced at 24 hours, the mechanical properties were not affected. It is postulated that a decrease in chondrocyte viability will lead to decreased production of collagen and other matrix proteins, eventually weakening the mechanical properties of the cartilage tissue. Therefore, a later assessment to allow for these cellular and matrix changes to develop may be required to detect any changes to the mechanical properties.

Articular chondrocytes have also been shown to be vulnerable to treatment with local anesthetics, including bupivacaine, *in vitro*. Chu and colleagues exposed bovine chondrocytes to 0.125%, 0.25%, and 0.5% bupivacaine for 15, 30, or 60 minutes and showed that viability showed a dose and time response to exposure, with the 0.125% exposure group showing viability similar to the control, the 0.25% exposure group showing an exposure time-related decrease in viability, and the 0.5% exposure group viability approaching 0% for all exposure times.^8^ Piper and Kim^9^ exposed human chondrocytes to 0.5% bupivacaine for 30 minutes and found that viability decreased to 37.4% of that of controls. A similar study by Grishko and colleagues that exposed primary human chondrocytes to 0.25% and 0.5% bupivacaine for 1 hour saw no significant decrease in viability at 24 hours post-exposure but a significant decrease for both exposure groups at 120 hours.^10^

This study aimed to determine the effects of a single bupivacaine exposure on cartilage explant mechanical properties at 3 weeks. The hypothesis was that the mechanical properties of cartilage explants would be weakened 3 weeks after single bupivacaine exposure and that this effect would be dose-dependent.

## Methods

### Explant Harvest

Cartilage punches, full-thickness and measuring 5 mm in diameter, were aseptically harvested from the femoral condyles and trochlea of 2 juvenile bovine stifle joints (Research 87) less than 36 hours after slaughter using 5-mm biopsy punches. Both specimens looked grossly normal without any apparent defects or abnormalities of the articular cartilage. Explants were trimmed to a thickness of 1 to 2 mm to include the articular surface, superficial zone, and part of the middle zone and were preserved in chondrogenic medium overnight before exposure.

### Bupivacaine Exposure

The explants were randomly divided into 3 groups: 0.50% bupivacaine (*n* = 8), 0.25% bupivacaine (*n* = 8), and chondrogenic medium with no bupivacaine (control, *n* = 7). Each explant was placed into its own well in a 12-well plate and submerged in 1.2 mL of its respective medium. This 1.2 mL was made up of 0.719 mL of 0.50% bupivacaine, 0.25% bupivacaine, or chondrogenic medium and mixed with 0.481 mL of chondrogenic medium to account for how a 10-mL injection would be diluted by the 6.7 mL of synovial fluid present in the human knee.^11^ The explants were placed on a shaker at 37 °C and 50 rpm for 1 hour to simulate single injection in the joint while accounting for synovial fluid absorption and leakage. After exposure, the explants were rinsed 3 times with chondrogenic medium, placed in a clean 24-well plate in fresh chondrogenic medium, and stored at 37 °C and 10% CO_2_. Fresh chondrogenic medium was exchanged every 2 days for 3 weeks until testing.

### Gross Morphology

Before testing, the explants were weighed and photographed. Wet weights were recorded, and photographs of the articular surface and the lateral face were taken. Thickness was measured at the center of the lateral face of each explant using ImageJ (National Institutes of Health).

### Viability

**Figure 1.**
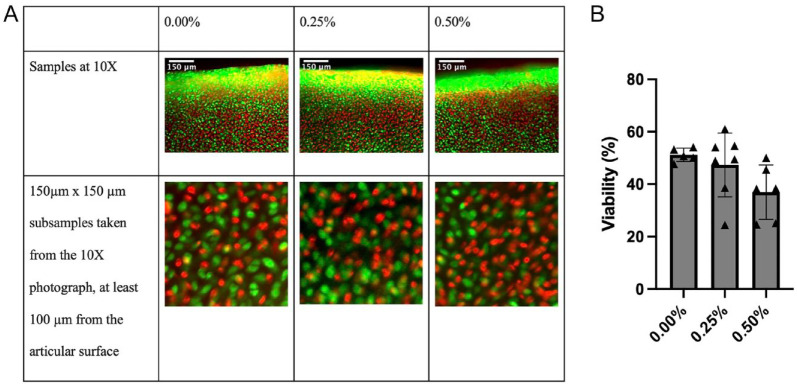
(**A**) Examples of the 10x overlaid photographs (upper row) and the 150 μm × 150 μm subsamples (lower row) used to count live (green) and dead (red) cells for each explant. The articular surface is at the top of the larger photos. The smaller images are 150-μm squares taken from at least 100 μm deep to the articular surface. (**B**) ImageJ was used to analyze photographs of the samples and count live and dead cells 3 weeks after bupivacaine exposure (examples shown in **Fig. 1A**). Three measurements were taken from non-overlapping regions for each explant and averaged to get 1 final viability value for each explant. Viability was 51.2 ± 2.57% for the control group, 47.3 ± 12.2% for the 0.25% bupivacaine-exposed group, and 37.0 ± 10.4% for the 0.50% bupivacaine-exposed group (*P* = 0.072).

A full-thickness, full-diameter, 1-mm-wide center strip was cut out of each explant for viability testing. The LIVE/DEAD reagent (calcein acetoxymethyl, BOBO-3 iodide; Thermo Fisher, R37601, Waltham, MA) was prepared as directed. The subsample was then submerged for 30 minutes in 80 μL of LIVE/DEAD reagent mixed with 80 μL of chondrogenic medium before being examined with light microscopy at 10x and 20x. Three non-overlapping regions of each sample were photographed with the Texas Red laser and the green fluorescent protein laser to visualize dead and live cells, respectively, with 6 photos per sample. Each region’s live and dead images were then overlaid using ImageJ software to create 3 composite images from non-overlapping areas per sample (**Fig. 1A**). ImageJ was then used to take a 150-μm × 150-μm square subregion from each composite, at least 100 μm in from the articular surface (**Fig. 1A**). A macro was then employed to count each subregion’s individual live and dead cells using the auto local threshold, watershed, and analyze particles functions. This resulted in 3 measurements per sample, averaged to get 1 total viability value for each sample.

### Biomechanical Testing

#### Compression

A 2-mm-diameter punch subsample was taken from each larger explant to be used in compression testing. A compression indentation apparatus (CIA)^12^ was used to measure the shear modulus, aggregate modulus, and permeability of the cartilage tissue, as previously described.^13^ The sample was placed under a 0.55-mm diameter, flat-ended, rigid, porous indenter tip, subjected to a tare weight between 4.5 and 10.5 g, and allowed to reach creep equilibrium at 10% strain. The CIA used a finite element analysis and a linear biphasic model to calculate the compressive properties.^12^ ImageJ software was used to measure the thickness of the samples from photos taken before testing, and this thickness was used for analysis.

#### Tensile

A dog bone–shaped subsample was taken from each explant for tensile testing, as has been previously described.^14^ The subsample was glued to a paper frame with a 0.8 mm gauge length for gripping, loaded into an Instron model 5565 (Instron, Canton, MA), and subjected to uniaxial tension using a 5-kN load cell until sample failure. The strain rate was set at 1% of the gauge length per second. The machine returned force-displacement curves. ImageJ software was used to measure the cross-sectional area of the smallest portion of the dog bone shape based on photos taken prior to tensile testing. This known area was used for creating stress-strain curves and calculating tensile Young’s modulus and ultimate tensile strength (UTS).

### Biochemistry

A subsample of each explant with an approximate wet weight of 2.5 mg was lyophilized and digested in 125 mg/mL papain (Sigma, St. Louis, MO) in phosphate buffer at 60 °C for 18 hours. Collagen content was measured using a modified colorimetric chloramine-T hydroxyproline assay with a Sircol collagen assay standard (Biocolor Ltd, Carrickfergus, United Kingdom).^15^ Sulfated GAG content was assessed using the Blyscan dimethyl methylene blue assay kit (Biocolor Ltd). Finally, DNA content was quantified with a PicoGreen cell proliferation assay (Quant-iT PicoGreen dsDNA assay kit; Thermo Fisher). Collagen and GAG content were normalized by wet weight, dry weight, and DNA content. DNA content was normalized by wet weight and dry weight.

### Collagen Crosslinks

The crosslinking assay was performed as previously described.^16^ A subsample of roughly 1 mg wet weight was taken from every sample. These were then digested in 100 μL of 6N hydrochloric acid at 105 °C for 20 hours, dried, and resuspended in 200 μL of diluent before being run through the mass spectrometer to measure the pyridinoline crosslink and hydroxyproline (OHP) content. Dry weight was calculated using the water content determined from the lyophilized samples for the other biochemical assays. Pyridinoline content was normalized by wet weight and OHP content. OHP content was normalized by wet weight.

### Histology

Small subsamples from each explant were fixed in 10% neutral buffered formalin and embedded in paraffin. Cross-sections 6-mm-thick were cut and placed on slides before being stained with hematoxylin and eosin to visualize cell morphology, safranin O to examine GAG distribution, and picrosirius red to evaluate collagen distribution within the tissue.^17^ Picrosirius red–stained samples were also visualized using polarized microscopy to determine whether there were apparent directional organization differences of collagen fibers within the samples.

### Statistics

Statistics were performed using GraphPad Prism 9 software (GraphPad Software Inc., San Diego, CA). A sample size of at least *n* = 6 was set based on a power analysis using previous mechanical data with alpha set at 0.05 and power set to 80%.^2,18^ Outliers were calculated using the GraphPad ESD method and excluded prior to running the analysis. Descriptive statistics were run to obtain each group’s mean and standard deviation. An analysis of variance (ANOVA) with Tukey’s *post hoc* test was used to determine any differences caused by bupivacaine dose. Significance was set at *P* < 0.05.

## Results

### Gross Morphology

There were no apparent differences in gross explant morphology among groups.

### Viability

Bupivacaine exposure had a dose-dependent chondrotoxic effect on the cartilage explants, but these differences were not significant. Three weeks after exposure, the control group had a viability of 51.2 ± 2.57%, the 0.25% exposure group had a viability of 47.3 ± 12.2%, and the 0.50% exposure group had a viability of 37.0 ± 10.4% (*P* = 0.072) (**Fig. 1B**).

### Biomechanical Testing

#### Compression

**Figure 2.**
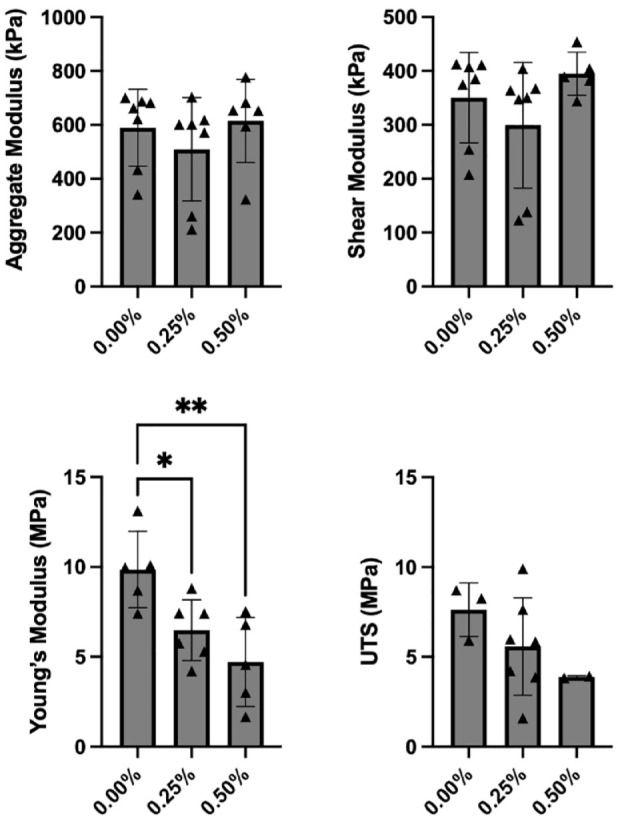
Biomechanical properties of explants 3 weeks after bupivacaine exposure. The aggregate modulus (upper left) was 590 ± 143 kPa for the control group, 510 ± 192 kPa for the 0.25% bupivacaine-exposed group, and 614 kPa ± 154 kPa for the 0.50% bupivacaine-exposed group (*P* = 0.495). Shear modulus (upper right) was 350 ± 83.8 kPa for the control group, 299 ± 117 kPa for the 0.25% bupivacaine-exposed group, and 395 ± 39.9 kPa for the 0.50% bupivacaine-exposed group (*P* = 0.219). Tensile Young’s modulus (bottom left) was 9.86 ± 2.12 MPa for the control group, 6.48 ± 1.69 MPa for the 0.25% exposure group (*P* = 0.048), and 4.72 ± 2.47 MPa for the 0.50% exposure group (*P* = 0.005). Ultimate tensile strength (UTS) (bottom right) was 7.63 ± 1.49 MPa in the control group, 5.58 ± 2.71 MPa in the 0.25% exposure group, and 3.88 ± 0.064 MPa in the 0.50% exposure group (*P* = 0.245). UTS = ultimate tensile strength. *P < 0.05, **P < 0.01.

Bupivacaine exposure had no significant effect on either the aggregate modulus (*P* = 0.495) or the shear modulus (*P* = 0.219) of the cartilage explants. For the control group, the aggregate modulus and shear modulus were 590 ± 143 kPa and 350 ± 83.8 kPa, respectively. For the 0.25% exposure group, the aggregate modulus and shear modulus were 510 ± 192 kPa and 299 ± 117 kPa, respectively. For the 0.50% exposure group, the aggregate modulus and shear modulus were 614 ± 154 kPa and 395 ± 39.9 kPa, respectively (**Fig. 2**).

#### Tensile

Both 0.25% and 0.50% bupivacaine exposure significantly decreased tensile Young’s modulus in cartilage explants compared with controls. Tensile Young’s modulus was 9.86 ± 2.12 MPa for the control group, 6.48 ± 1.69 MPa for the 0.25% exposure group (*P* = 0.048), and 4.72 ± 2.47 MPa for the 0.50% exposure group (*P* = 0.005) (**Fig. 2**). Although there was a similar dose-dependent decrease in UTS, these differences were not significant. UTS was 7.63 ± 1.49 MPa in the control group, 5.58 ± 2.71 MPa in the 0.25% exposure group, and 3.88 ± 0.064 MPa in the 0.50% exposure group (*P* = 0.245) (**Fig. 2**).

### Quantitative Biochemistry

Bupivacaine exposure did not affect DNA or collagen content of the cartilage explants when measured with the benchtop assay. DNA content was 0.017 ± 0.002% (DNA/wet weight) for the control group, 0.018 ± 0.003% for the 0.25% exposure group, and 0.019 ± 0.002% for the 0.50% exposure group (*P* = 0.355). Collagen content was 10.1 ± 1.67% (collagen/wet weight) for the control group, 9.04 ± 0.788% for the 0.25% exposure group, and 9.36 ± 1.38% for the 0.50% exposure group (*P* = 0.349). GAG content was increased for the 0.25% exposure group relative to controls. GAG content was 6.72 ± 0.311% (GAG/wet weight) for the control group, 7.86 ± 0.981% for the 0.25% exposure group (*P* = 0.036), and 7.14 ± 0.903% for the 0.50% exposure group (*P* = 0.611).

**Figure 3.**
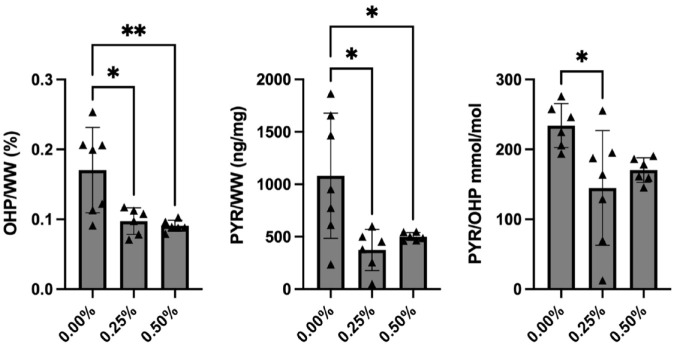
Mass spectrometry results for collagen content (using OHP as a proxy) and collagen crosslinking (using pyridinoline as a proxy). OHP/wet weight (left) was 0.170 ± 0.061% for the control group, 0.097 ± 0.019% for the 0.25% exposure group (*P* = 0.011), and 0.091 ± 0.008% for the 0.50% exposure group (*P* = 0.006). Pyridinoline/wet weight (middle) was 0.108 ± 0.060% for the control group, 0.037 ± 0.020% for the 0.25% exposure group (*P* = 0.011), and 0.050 ± 0.004% for the 0.50% exposure group (*P* = 0.038). Pyridinoline/OHP (right) was 234 ± 31.5 mmol/mol for the control group, 145 ± 82.1 mmol/mol for the 0.25% exposure group (*P* = 0.023), and 170 ± 17.6 for the 0.50% exposure group (*P* = 0.135). OHP = hydroxyproline. *P < 0.05, **P < 0.01.

Conversely, the mass spectrometry assay using hydroxyproline as a marker of collagen showed statistically significant decreases in collagen content for both the 0.25% and the 0.50% exposure groups. OHP/wet weight was 0.170 ± 0.061% for the control group, 0.097 ± 0.019% for the 0.25% exposure group (*P* = 0.011), and 0.091 ± 0.008% for the 0.50% exposure group (*P* = 0.006) (**Fig. 3**). Pyridinoline content also decreased with both 0.25% and 0.50% bupivacaine exposure. Pyridinoline content was 0.108 ± 0.060% (pyridinoline/wet weight) for the control group, 0.037 ± 0.020% for the 0.25% exposure group (*P* = 0.011), and 0.050 ± 0.004% for the 0.50% exposure group (*P* = 0.038) (**Fig. 3**). When controlled for OHP content, there was a significant decrease in PYR/OHP for the 0.25% bupivacaine exposure group relative to the control group. PYR/OHP was 234 ± 31.5 mmol/mol for the control group, 145 ± 82.1 mmol/mol for the 0.25% exposure group (*P* = 0.023), and 170 ± 17.6 for the 0.50% exposure group (*P* = 0.135) (**Fig. 3**).

### Histology

**Figure 4.**
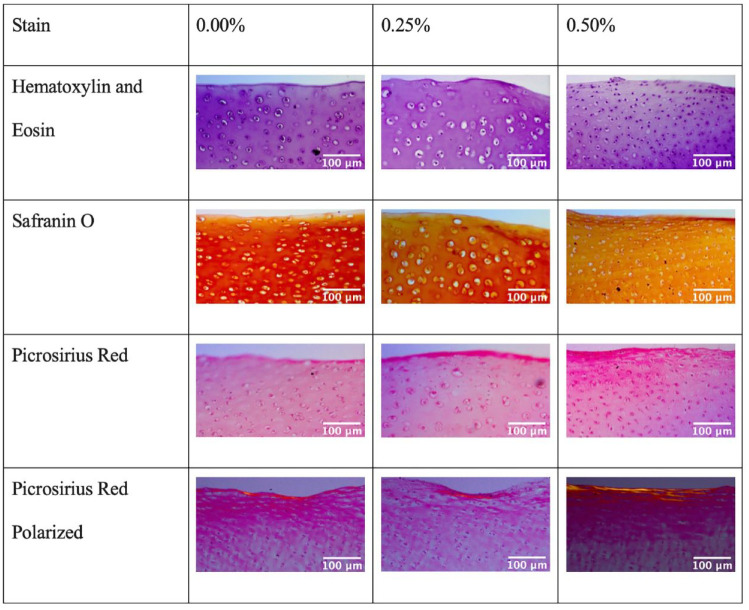
Examples of histology photographs for each exposure group and staining. There were no apparent differences visible with histology when comparing groups.

Bupivacaine exposure did not affect the histological appearance of cartilage explants in any of the 3 stains (picrosirius red, safranin O, or hematoxylin and eosin) (**Fig. 4**). The morphology of the cells, distribution of collagen, distribution of GAG, and general appearance of the tissue appeared unchanged among exposure groups. Similarly, examining the picrosirius red–stained samples under polarized microscopy did not reveal differences in the directional organization of collagen fibers within the samples among the different groups (**Fig. 4**).

## Discussion

This study found that a single, hour-long exposure to bupivacaine decreased the tensile properties (Young’s modulus) of bovine cartilage explants 3 weeks after exposure. Bupivacaine exposure was also associated with reduced collagen and pyridinoline content by mass spectrometry, consistent with decreased tensile properties. There were no significant differences in the compressive properties, viability, or histological appearance among exposure groups.

Several studies have historically shown that bupivacaine and other local anesthetics are chondrotoxic, both with increasing dosage and time of exposure.^2,4,19^ Although there was a dose-dependent chondrotoxic trend on the cartilage explants in this study, these differences were not statistically significant. This finding could be attributed to several factors, including prolonged *in vitro* culture. Keeping the *in vitro* explants in culture for 3 weeks likely decreases the overall viability due to inherent external stressors with culture, and any differences attributed to bupivacaine exposure could be attenuated. Another factor may be that some of the effects of bupivacaine exposure on viability decrease with time as the tissue is given more time to recover. This is less likely considering the slow turnover of cartilage and the senescence of chondrocytes embedded within the cartilage matrix.^20^ With these low viability numbers in the controls at 3 weeks post-exposure, there is some question as to where the critical border lies for such an experiment. While we did not measure viability of the samples at 1 or 2 weeks post-exposure, Chu and colleagues exposed chondrocytes to 0.25% bupivacaine for 15, 30, or 60 minutes and tested viability at 1 hour, 24 hours, and 1 week and found that the control remained stable around 65% viability at all time points while there was an exposure time decrease in viability that became more significant at later time points.^8^ This, combined with Oyadomari’s results, suggests that it may be logical to further explore the timeframe between 1 and 3 weeks post-exposure.^2^ Models that better replicate the *in vivo* condition are likely better suited to explore the magnitude of chondrotoxicity from single bupivacaine exposure.

There are currently limited data on how bupivacaine exposure affects the mechanical properties of cartilage, especially over time. From a mechanical perspective, the tensile strength of cartilage stems from its collagen content, organization, and crosslinking. In this study, both collagen content and crosslinking were decreased through the hydroxyproline and pyridinoline content mass spectrometer assays. In contrast, collagen organization was not affected as measured via picrosirius red histology. Hydroxyproline is a valuable marker for collagen content, and it makes up around 13.5% of the protein.^21^ It is thought to stabilize collagen and is frequently found as the Y in the triplet Gly-X-Y.^21-23^ Pyridinoline crosslinks are also known to increase the tensile strength in cartilage, including the knee meniscus.^14,24,25^ The effect of the decrease in hydroxyproline was likely compounded by the decrease in pyridinoline, meaning that not only was there less collagen in the bupivacaine-exposed samples, but the existing collagen had fewer crosslinks. This is consistent with a bovine study that found that tensile stiffness was more dependent on crosslink density than collagen content.^14^ In the native joint, the articular cartilage is exposed to tensile, compressive, and shear stresses. The decrease in tensile stiffness from bupivacaine may predispose to further matrix damage and fracture of the cartilage tissue in response to tensile deformation.^26^

The short-term study performed by Oyadomari and colleagues found no effect of bupivacaine exposure on compressive mechanical properties 24 hours after exposure.^2^ It was hypothesized that a longer incubation period could allow damage from bupivacaine exposure to extend to the compressive properties of explants. However, that was not seen in this study and suggests that either such effects may occur earlier than 3 weeks and be transient, these hypothetical decreases need longer than 3 weeks to appear, or single bupivacaine exposure may not affect the compressive properties at all. The values for both aggregate modulus and shear modulus were about twice as high in this study as in Oyadomari’s investigation, which is also in line with the higher GAG content observed in all 3 groups. The GAGs in cartilage tissue limit water flow out of the tissue, allowing it to withstand compressive loads.^27^ Although GAG per wet weight was higher for the 0.25% bupivacaine group than for the control group in this study, this difference was slight and not borne by any changes in the compressive properties measured.

In addition, extensive research has been done in recent years about the role that senescence plays in the development of osteoarthritis (OA) and joint degeneration in general. It has been shown that the concentration of senescent chondrocytes in cartilage increases with age, and hypothesized that this change contributes to the development of OA.^28^ It has also been shown that factors such as mechanical loading, specific chemokines, cytokines, and proteases increased oxidative stress, metabolic syndrome, and autophagy from stressful environments can increase senescence.^29-31^ While we did not quantify senescence in this study, it is worth noting that many factors known to increase senescence were likely present in these samples. It would be valuable to further explore these effects in future studies.

This study has several limitations. First, compared with the *in vitro* model used in this study, experimental models that better simulate the *in vivo* condition are needed to explore the effects of bupivacaine on the mechanical properties of articular cartilage. In addition, this study used bovine cartilage, so no extrapolation can be made to other species, including humans, without verifying the same effects in those species. The bupivacaine exposure protocol used in this study may have been shorter than an *in vivo* injection, as the half-life of bupivacaine is longer than the 1-hour exposure time this study used.^2^ Finally, there were discrepancies in the significance of the collagen results between the benchtop and mass spectrometry assays.

## Conclusion

Three weeks after 1-hour bupivacaine exposure, the tensile properties of bovine cartilage explants were significantly decreased, while the compressive properties remained unaffected. These decreases in tensile properties corresponded with reductions in collagen content and crosslinking of collagen fibers.
